# Prokinetic effect of erythromycin in the management of gastroparesis in critically ill patients—our experience and literature review

**DOI:** 10.3389/fmed.2024.1440992

**Published:** 2024-09-09

**Authors:** Mateusz Szczupak, Magdalena Jankowska, Bartłomiej Jankowski, Jolanta Wierzchowska, Jacek Kobak, Paweł Szczupak, Justyna Kosydar-Bochenek, Sabina Krupa-Nurcek

**Affiliations:** ^1^Department of Anesthesiology and Intensive Care, Copernicus Hospital, Gdansk, Poland; ^2^Department of Otolaryngology, Medical University of Gdansk, Gdansk, Poland; ^3^Department of Electrical Engineering and Computer Science, Rzeszow University of Technology, Rzeszow, Poland; ^4^Institute of Health Sciences, College of Medical Sciences of the University of Rzeszow, Rzeszow, Poland; ^5^Department of Surgery, Institute of Medical Sciences, Medical College of Rzeszow University, Rzeszow, Poland

**Keywords:** gastroparesis, gastrointestinal paralysis, erythromycin, critically ill patient, intensive care

## Abstract

**Introduction:**

Gastroparesis is a disorder characterized by impaired gastric emptying and the accumulation of food in the intestines without any clear mechanical cause. Gastroparesis in critical care patients is a prevalent issue in the intensive care unit. The disruption of normal gastrointestinal motility in critically ill patients is linked to a significant risk of intolerance to enteral feeding, colonization of the gastrointestinal tract with pathogenic bacterial strains, increased permeability of the intestinal wall, translocation of the intestinal microbiota, leading to progressive malnutrition, and potential development of bacterial infection.

**Materials and methods:**

The literature was reviewed to assess the benefits and risks associated with the use of this medication.

**Aim:**

The aim of the study was to treat the symptoms of gastroparesis and stimulate gastrointestinal motility. Consequently, the aim was to reduce the amount of backed-up food content in the stomach, accelerate gastrointestinal motility, and return to intestinal feeding.

**Results:**

Gastroparesis is a frequent issue among patients in the intensive care unit. Critical illness can lead to gastrointestinal motility disorders, causing slowed gastric emptying. This increases the risk of problems such as intolerance to enteral feeding, regurgitation, and aspiration of gastrointestinal contents into the respiratory tract, as well as colonization of the gastrointestinal tract by pathogens. Over time, impaired intestinal absorption can result in malnutrition, necessitating the initiation of parenteral nutrition.

**Conclusion:**

After analysis of the literature and published scientific reports, as well as considering their own research, it is evident that erythromycin, as a prokinetic drug, effectively enhances gastrointestinal motility. This contributes to stimulating gastric emptying in critically ill patients with gastroparesis who are hospitalized in an intensive care unit. The use of erythromycin in combination with metoclopramide and/or itopride hydrochloride allows for a synergistic effect, leading to the quickest possible return to enteral feeding.

## 1 Introduction

Gastroparesis, also known as gastrointestinal paralysis, is a disorder characterized by impaired gastric emptying and the accumulation of food in the intestines without any clear mechanical cause (1, 2). Common clinical symptoms include nausea, vomiting, early satiety, postprandial fullness, bloating, or upper abdominal pain, which typically occur in conscious patients and are often the result of neurological and endocrine disorders (3–5). Gastroparesis in critical care patients is a prevalent issue in the intensive care unit. The disruption of normal gastrointestinal motility in critically ill patients is linked to a significant risk of intolerance to enteral feeding, colonization of the gastrointestinal tract with pathogenic bacterial strains, increased permeability of the intestinal wall, translocation of the intestinal microbiota, leading to progressive malnutrition, and potential development of bacterial infection.

Reintam et al. estimated that at least 60% of patients hospitalized in the intensive care unit are affected by gastrointestinal dysfunction (6). In contrast, Gungabissoon et al. (7) reported that 30% of critically ill patients need to be switched from enteral to parenteral forms of nutrition due to intolerance.

The severity of gastric motility disorder in patients with gastroparesis does not always match the severity of their symptoms. The severity of gastric motility disorder in patients with gastroparesis does not always match the severity of symptoms. According to the literature, this difference may be due to the diagnostic methods used (8–11). The most common causes affecting normal gastric function include hyperglycemia, damage to the vagus nerve, and drugs that inhibit gastrointestinal peristalsis, such as opioids. According to Chawla et al., (12) hyperglycemia occurs in 49.8% of critically ill patients. The authors also note that hyperglycemia occurs in 43.7% of critically ill patients with heart failure. On the other hand, causes of gastroparesis from surgical intestinal motility in intensive care unit patients include impaired perfusion of the gastrointestinal wall, release of pro-inflammatory cytokines in sepsis, edema of the intestinal wall due to capillary leakage, disturbances in the secretion of hormones responsible for regulating motility, sedatives or vasopressors, and mechanical ventilation (13, 14).

The consequence of gastroparesis in critical ill patients is the inability to provide the patient with enough calories, which can lead to malnutrition. This can result in prolonged hospitalization in the intensive care unit, excessive gastric distension, the possible development of intra-abdominal tightness syndrome, and an increased risk of mortality (5, 15).

Strojek and Jasinski’s paper discusses the challenges in defining and establishing criteria for differentiating the mechanisms that cause gastroparesis. They conducted a systematic review of Blaser et al.’s work and found that Blaser identified 43 definitions of gastroparesis, many of which are based on assessing the volume of food retained in the stomach with varying values for this parameter (1). Blaser et al. (5) noted the difficulty in accurately estimating the prevalence of gastroparesis in patients in the intensive care unit. In their opinion, this is related to the different definitions of gastric backlog volume.

Assessment of gastric emptying is determined by measuring the backlog volume. Chapman et al. (16) suggest that a backlog volume of more than 150 mL over 24 h indicates impaired gastric emptying, which requires intervention. On the other hand, Strojek and Jasinski emphasize that the backlog volume can range from 75 to 500 mL, and measuring it is controversial due to the lack of standardization and the influence of confounding factors (1). Metheny et al. (17) point out that the measured backlog volume is affected by the diameter of the gavage, the aspiration technique, the density of the administered food, the patient’s body position, and other factors.

The gold standard for diagnosing gastroparesis is scintigraphy, which is typically performed on outpatients but can also be done in the intensive care unit. However, it is currently not practical for intensive care units due to its time-consuming nature. Willems et al. suggest that evaluating paracetamol absorption could be a useful method for assessing gastric emptying. Paracetamol is absorbed in the small intestine. When taken orally and its concentration in the blood is measured, it can help determine the rate of gastric emptying. However, because the test is not standardized and is time-consuming, this method is not currently used in clinical practice (18).

## 2 Aim of the study

In this review, we discussed the pathophysiology of gastroparesis, current diagnostic standards, and the characteristics of erythromycin as a prokinetic drug according to the literature. In addition, we describe the experience of our service in the treatment of the symptoms of gastroparesis and stimulate gastrointestinal motility, in order to reduce the amount of backed-up food content in the stomach, accelerate gastrointestinal motility, and return to intestinal feedin.

## 3 Materials and methods

**Figure 1:**
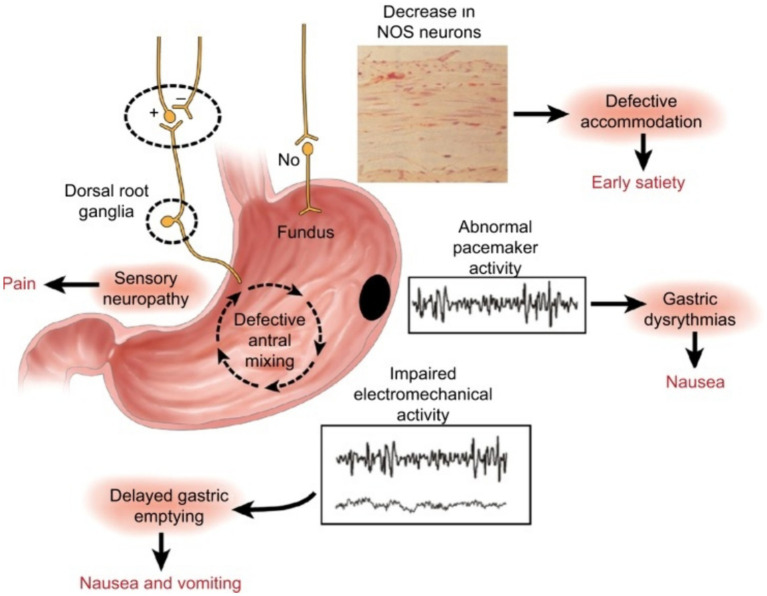
Summary of the neural, myoelectrical, muscular, and cellular aspects of the pathophysiology of gastroparesis. Image source: https://www.ncbi.nlm.nih.gov/pmc/articles/PMC6165730/.

To write this manuscript, we reviewed articles available on PubMed, Google Scholar and Mendeley. We used keywords like erythromycin, gastroparesis, and gastrointestinal motility disorder to find relevant publications. From the articles we found, we selected those that discussed the prokinetic effect of erythromycin and were considered interesting sources of information by the authors. We analyzed a total of 99 selected manuscripts, from which 74 were cited in this paper. Regarding scientific reports indicating the prokinetic effect of erythromycin, the authors of the manuscript use this drug as one of the treatments for gastroparesis in their daily work. This treatment is used in critical care conditions and was observed to confirm previous findings about the prokinetic effect of erythromycin by other authors (Figure 1).

## 4 Pathophysiology, diagnosis, and current treatment options

### 4.1 Pathophysiology

The mechanism of gastroparesis is complex. It most likely begins with impaired microcirculation in the gastric wall (19). Damage to the gastric neurons responsible for nitric oxide (NO) synthesis impairs the pathway of its metabolism in the intestinal myenteric plexus, leading to impaired gastric accommodation reflex and late dyspeptic symptoms (20). The development of gastroparesis is associated with damage to intrinsic or extrinsic neurons, Cajal’s interstitial cells, or loss of heme oxidase 1 (20–22). This damage can result in delayed gastric emptying due to the stomach’s inability to relax and expand, excessive contraction of the opening between the stomach and small intestine, reduced ability of the muscles in the area just before the opening to contract, or problems with coordination of muscle movement between the stomach and small intestine (20). Patients with gastroparesis also exhibit an altered ratio of pro-inflammatory and anti-inflammatory macrophages (23–25). This involves reducing CD206-positive anti-inflammatory macrophages (M2) in the body’s circular muscles and gastric antrum (23, 26–28).

The causes of gastroparesis can include cancer, such as small cell lung cancer, paraneoplastic syndromes, chemotherapy, radiation therapy, and surgical procedures like fundoplication, bariatric surgery, or pancreatic resection. Other possible causes of gastric motility paralysis may involve systemic scleroderma, systemic mastocytosis, systemic lupus erythematosus, or amyloidosis (20, 29–31).

### 4.2 Diagnosis

The diagnosis of gastroparesis is confirmed based on typical clinical symptoms along with objectively established delayed gastric emptying, without a mechanical cause. To start, an upper gastrointestinal tract examination (gastroscopy) is conducted. The gold standard for diagnosing gastroparesis is scintigraphic evaluation of gastric emptying. An abnormal scintigraphy result is indicated by a backlog of 10% of the volume of food labeled with radioactive technetium 4 h after ingestion (20, 32). Another diagnostic method that correlates well with scintigraphy is a wireless capsule designed to assess pH levels in the surrounding environment. When the capsule leaves the stomach, there is an immediate increase in pH value. This method allows for assessing the motility of the stomach and other parts of the gastrointestinal tract. According to Lee et al., gastric emptying can be delayed if the capsule reaches the duodenum more than 5 h after ingestion (32, 33). However, it’s important to note, as mentioned by Cassilly et al. in their paper, that undigested food particles, including the capsule, may pass through with a delay. This delay may not accurately reflect the actual gastric emptying time (33).

The Table 1 outlines the methods for diagnosing gastroparesis, including symptoms suggestive of its presence and a description of the expected test result.

**Table 1:** The diagnostic methods used to diagnose gastroparesis.

Diagnostic methods	Symptoms suggestive of gastroparesis	Discription of the study
X-ray examination with barium administration	Stomach dilation and reduced peristaltic movement.	Invasive method. Barium is used to assess the morphology and motility of the gastrointestinal tract (68).
Ultrasound examination and Doppler techniques	Evaluate the changes in the antral segment of the stomach after consuming liquid meals, which correlates with the rate of gastric emptying.	A less invasive method than X-ray. Provides a better evaluation of morphology.
Three-dimensional ultrasonography		It provides more comprehensive information about gastric function than traditional two-dimensional examinations. This method is time-consuming and requires experience and specialized equipment (69).
Radioisotope scintigraphy	Delayed gastric emptying: There is no significant difference in the emptying of solid foods compared to liquid foods.	The gold standard for diagnosing gastroparesis involves measuring the rate of gastric emptying using a meal enriched with technetium 99 isotope (68). The meal’s composition and caloric content are standardized. If the emptying time is prolonged by more than 10% over the 4-h standard for men, it is considered a positive result. For women, the prolongation must exceed 20% to be considered a positive result (70).
Exhaust test	Assessment of labeled carbon dioxide in expiratory air correlates with gastric emptying rate.	This test is used for rapid diagnostics. It involves enriching food with the C13 carbon isotope and measuring the time of its excretion in the carbon monoxide molecule (68). The test has a specificity of 80% and a sensitivity of 86%. However, this test is not specific in patients with concomitant lung, pancreatic, liver disease, and short bowel syndrome, especially in cases of very severe gastroparesis (70).
Manometry	Reduced stomach muscle movement, along with an abnormal extended contraction of the pyloric sphincter.	The study assesses how often and how strongly the stomach contracts when empty, after a meal, and after taking prokinetic drugs. Gastroparesis is characterized by a lack of normal muscle movement between meals, weaker contractions after eating, and in some cases, a complete absence of muscle movement in both the stomach and the duodenum (71).
Electrogastrography	Myoelectric dysfunction.	It enables the evaluation of gastrointestinal motor function, allowing for the simultaneous differentiation between neuropathy and myopathy (68).
Capsule endoscopy	The pH changes from acidic in the stomach to alkaline in the duodenum.	The evaluation of gastric emptying involves assessing the shift in pH from acidic in the stomach to alkaline in the duodenum. This test has a sensitivity of 87% and a specificity of 92%. Furthermore, pressure measurements offer additional insights into the motor functions of the stomach, small intestine, and large intestine (69).

### 4.3 Current treatment options

Currently, treatment options for gastroparesis include nutritional, pharmacological, and non-pharmacological management. Table 2 presents the various treatments for gastroparesis and their results.

**Table 2:** Current treatments for gastroparesis.

Methods	Results		
Nutritional treatment	Reduce the severity of sadness and prevent nutritional deficiencies by adjusting the diet, including reducing meal sizes, limiting fat intake, and consuming food in smaller portions. Enteral or parenteral nutrition may be necessary in severe cases.		
Discontinuation of medications that slow down digestion like opioids, antycholinergic, GLP-1 agonists	Improvement of gastrointestinal motility.		
Pharmacological treatment	Prokinetic drugs	Dopamine D2 receptor antagonists	Domperidon 10 mg 3 times per day before meals
			Itopride 50 mg 3 times per day
		Serotonin 5-HT4 receptor agonists	Cisapride 10 mg 3–4 times per day, 15 min before meals
			Prucalopride
		D2 receptor antagonist/ 5-HT4 receptor agonist	Metoclopramide 10 mg 1–3 times per day
		Motilin receptor agonists	Erythromycin 100–200 mg 2–3 times per day before meals. It will be using the mask for 4 weeks because of the tachyphylaxis phenomenon.
	Medications that decrease nausea and prevent vomiting	5-TH3 receptor antagonists	Ondansetron, granisetron
		Phenothiazine derivatives	Prochloropernazine, tiethylpernazine
		Antihistaminic drugs	Dimenhydrinate
Gastric electrical stymulation	Reduce the frequency of vomiting, especially in patients with diabetic gastropathy		
Pyloric invasion procedures	Endoscopic pyloromyotomy, laparoscopic or surgical pyloromiotomy/pyloroplastic, botulinum toxine		
Gastrectomy	To consider in very severe cases		

## 5 Prokinetic drugs

Prokinetics are medications that affect complex neurohumoral mechanisms. They work by stimulating coordinated contractions of the gastrointestinal muscles, leading to increased tone in the lower esophageal sphincter, faster gastric emptying, and shorter intestinal transit time (34–36). Their primary use is for conditions that inhibit or disrupt gastrointestinal motility. Prokinetics are also used in functional constipation, irritable bowel syndrome with constipation, before gastroscopy in patients with upper gastrointestinal bleeding, or patients with enteral feeding failure (37).

## 6 Erythromycin as a prokinetic drug

### 6.1 Characteristics and discovery of erythromycin

Erythromycin is a type of antibiotic belonging to the macrolide group, which was discovered in 1952. It is made up of a combination of compounds, with the active macrolide component being Streptoerythromycin A, containing a 14-membered macrocyclic lactone ring (38). Its mechanism of action involves blocking protein biosynthesis by reversibly binding to the 50S subunit of the ribosome and disrupting the elongation of the polypeptide chain (39). It is an antibiotic that has antimicrobial activity against both Gram-positive and Gram-negative bacteria. It is used to treat tonsillitis, acute sinusitis, pneumonia, and other infections. The side effects of this drug, such as improved gastric motility, were first described in 1984 (40). Erythromycin has motilin-like effects on gastric muscle. It binds to smooth muscle receptors and myenteric neurons (41). Depending on the dose, it can have different effects, as shown in cases of diabetic gastroparesis (42). Tack et al. (43) confirmed that the drug’s effects are dose-dependent. In contrast, Koutsoumbi et al. and Pennathur et al. (44, 45) demonstrated that a low dose of erythromycin not only enhances the motor activity of the stomach but also of the esophagus and duodenum. In several studies, researchers evaluated intravenous doses ranging from 40 to 500 mg. Boivin et al. and Nguyen et al. (46, 47) demonstrated that administering 200 mg of erythromycin intravenously over 20–30 min effectively impacts gastric motility in the intensive care unit. Ritz et al. (47) also found that a 70 mg intravenous dose of the drug is equally effective.

## 7 Own experience

Between August 1, 2023, and December 31, 2023, a pilot study was conducted to examine the prokinetic effect of erythromycin. During this period, a total of 142 patients were hospitalized. Symptoms of gastropathy were observed in 39 (27.5%) patients (18 women and 21 men), with 23 (16.2%) patients diagnosed with a critical state of gastropathy. Among 39 (27.5%) patients, 24 (16.9%) were surgical and 15 (10.6%) were non-surgical. Of the chronic diseases, 20 (14.1%) patients had diabetes and hypertension, and 10 (7%) had only diabetes. 15 (10.6%) patients had been treated with antidiabetic oral drugs and 5 (3.5%) with insulin. The ages of the patients ranged from 31 to 80 years old (average age 50 years). The length of hospitalization in the intensive care unit ranged from 8 to 15 days (median = 13; IQR = 4). Those affected required parenteral nutrition due to gastrointestinal motility dysfunction and were treated with prokinetic drugs as part of their therapy. The initial treatment involved receiving 10 mg of intravenous metoclopramide three times a day. When there was no improvement, itopride was added as a second-line treatment at a dose of 50 mg three times a day, administered sublingually alongside metoclopramide. Despite such treatment, no resolution of gastroparesis symptoms and the desired return of gastrointestinal function were observed in the group of patients diagnosed with critical gastroparesis. In 17 patients, a nasoduodenal tube was inserted under endoscopic guidance, and enteral nutrition was initiated to bypass the stomach. Among this group, 3 patients experienced the backflow of undigested food into the stomach due to gavage translocation.

In the next phase of treatment, 23 patients (10 women and 13 men) with gastroparesis of critical care were administered erythromycin at a dosage of 300 mg three times a day via intravenous infusion. The duration of erythromycin treatment ranged from 2 to 9 days (median = 5; IQR = 3). Among the patients, 20 received triple prokinetic therapy comprising erythromycin, metoclopramide, and itopride. Two patients were treated with a combination of erythromycin and metoclopramide, while one patient received simultaneous erythromycin and itopride.

In most patients diagnosed with critical state gastroparesis, the inclusion of erythromycin led to the resolution of gastroparesis symptoms and the return of gastrointestinal function as early as day 2 of therapy. This was clearly associated with accelerated gastric emptying. Therefore, the treatment regimen with erythromycin enabled a rapid return to enteral feeding through gastric delivery of an industrial diet. Only two patients did not achieve the expected clinical effect. Our team’s findings regarding the use of erythromycin as a prokinetic drug align with existing research. It’s important to note that using erythromycin as a dual prokinetic therapy allows for fast and effective restoration of gastric motility with minimal risk of side effects. Additionally, in our study group, we observed no cases of diarrhea associated with clostridium difficile infection as a result of broad-spectrum antibiotic therapy.

## 8 Discussion

Erythromycin, as a prokinetic drug, accelerates gastric emptying in both healthy individuals with normal gastrointestinal function and in patients with critical gastroparesis, including the secondary form after esophageal-gastric surgery (43, 48, 49). Yeo et al. conducted a randomized trial involving 118 patients undergoing pancreatic surgery to evaluate the effect of erythromycin in preventing postoperative gastroparesis. They found that the frequency of gastric motility disorders was significantly lower in the erythromycin-treated group compared to the control group (19% vs. 30%, *p* < 0.05). This study confirmed the benefit of intravenous erythromycin in preventing postoperative gastroparesis after pancreatoduodenectomy (50). Collard et al., in turn, evaluated the effect of a continuous intravenous infusion of erythromycin at a dose of 1 g/24 h starting at the time of surgery with subsequent oral continuation on gastrointestinal motility after high esophageal-gastric anastomosis. They proved its effectiveness in maintaining postoperative antral motility (51). Shaikh et al. confirmed the effectiveness of erythromycin as a prokinetic in postoperative intensive care patients. They conducted a study to determine the optimal dose (13). Stevens et al., like Ritz et al., also demonstrate in their review paper the efficacy of low doses of intravenous erythromycin in the treatment of gastroparesis (52). Additionally, as reported by numerous authors, the enteral form of erythromycin is easy to administer and has few side effects (53, 54). This is supported by Hirsch et al., who conducted a study on critically ill patients, and Taylor et al., who examined the use of improving gastric motility despite metoclopramide’s lack of effect with dual prokinetic treatment (55, 56).

In a prospective, randomized study, Lu et al. demonstrated that the combination of metoclopramide and intravenous erythromycin had superior prokinetic effects compared to when these drugs were used alone. The combination also resulted in fewer side effects in intensive care unit patients (57). Shah et al. (58) further confirmed the effectiveness of pulse therapy, which consists of a combination of metoclopramide and erythromycin, in patients with critical gastroparesis.

In a study conducted by Nguyen et al., 143 cases of patients who received a prokinetic dose of erythromycin for 7 days and subsequently developed diarrhea were analyzed. Upon microbiological examination, none of the patients showed the presence of *Clostridium difficile* spores. The hypothesis proposed to explain the absence of *Clostridium difficile* toxin development after erythromycin therapy is that the medication accelerates gastrointestinal transit, thereby preventing the colonization and growth of pathogenic bacteria in the gastrointestinal tract (59, 60).

Sanger et al. addressed the effects of motilin receptor agonists on gastrointestinal motility in one of their papers. They mentioned that erythromycin, while showing agonistic effects against the motilin receptors, also has a non-selective character as it can inhibit P2X purinergic receptors. The authors also discussed its effectiveness in rapidly removing gastric contents before endoscopic examination or surgical treatment, as well as its use in treating patients with gastroparesis or chronic pseudo-obstruction of the intestines. Additionally, erythromycin is used in the treatment of premature infants with food intolerance or patients requiring the facilitation of enteral feeding. However, the researchers note that the dose of the drug is generally lower than that used to treat bacterial infections. Despite its potential, the use of erythromycin remains limited due to the risk of inducing bacterial resistance to the drug and prolonging the QT interval in the ECG, which could result in cardiac arrest (61).

In the manuscript “Development of Drugs for Gastrointestinal Motor Disorders: Translating Science to Clinical Need,” Sanger and Alpers point out that erythromycin, despite being used outside of its registered indications, exerts a good prokinetic effect. The authors emphasize that despite this efficacy, the optimal dose of the drug that improves gastric emptying and does not cause serious side effects has not yet been established. In their paper, the researchers cite other scientific reports indicating the beneficial prokinetic effect of intravenously administered erythromycin in a variable dose (62).

In a document entitled “United European Gastroenterology (UEG) and European Society for Neurogastroenterology and Motility (ESMN) consensus on gastroparesis,” the authors discuss the definition of gastroparesis, its symptoms, how it develops, and how it’s diagnosed. They also present their stance on treatment methods and their effectiveness. They emphasize the effectiveness of prokinetic drugs like itopride, metoclopramide, and motilin receptor agonists, such as erythromycin, in treating gastroparesis (63).

In their paper “A North American Perspective on the ESNM Consensus Statement on Gastroparesis,” Camilleri et al. recommend using intravenous erythromycin to treat acute gastroparesis in hospitalized patients. The authors mention that azithromycin, a macrolide antibiotic, has a similar impact on improving gastric emptying as erythromycin but with fewer side effects. Additionally, they discuss the prokinetic effects of other drugs and emphasize that prokinetic therapy is the most appropriate first-line therapy for gastroparesis. They point out that prokinetic drugs not only alleviate gastroparesis symptoms but also speed up gastric emptying (64).

The appropriate prokinetic dose of erythromycin is still a matter of debate. In a review article, Sanger and Andrews analyze the pharmacological rationale for choosing drugs that can inhibit vomiting or increase gastric emptying in treating gastroparesis. They point out that the effectiveness of erythromycin in reducing gastroparesis symptoms is inconclusive, as it is based on small studies that have not determined the optimal dose of the drug (65).

According to Camilleri’s research, a prokinetic effect can be achieved by administering 250 mg of erythromycin once a day for a week (66). In contrast, Grant and Thomas suggest a dose of 50–100 mg, to be taken four times a day, which they claim results in a much better prokinetic effect (67). In our own experience, a dose of 300 mg taken three times a day has led to a significant improvement in gastrointestinal motility as early as the second day of use. This allowed the symptoms of gastroparesis to decrease, which enabled the resumption of enteral feeding.

## 9 Conclusion

After analysis of the literature and published scientific reports, as well as considering their own research, it is evident that erythromycin, as a prokinetic drug, effectively enhances gastrointestinal motility. This contributes to stimulating gastric emptying in critically ill patients with gastroparesis who are hospitalized in an intensive care unit. The use of erythromycin in combination with metoclopramide and/or itopride hydrochloride allows for a synergistic effect, leading to the quickest possible return to enteral feeding.

Despite being an antibiotic used to treat bacterial infections, this drug, like other antibiotics, has the potential for side effects. When used as a prokinetic mechanism in patients hospitalized in the intensive care unit, who are often receiving multiple drug therapies, including broad-spectrum antibiotics, there is a risk of developing clostridium difficile infection. However, both the observations of the authors of this article and the literature reports support the idea that this drug has an effective prokinetic effect with a relatively low risk of side effects.

Gastroparesis is a common problem among intensive care unit patients. If ineffectively treated, it contributes to a worsening of the patient’s prognosis and prolongs hospitalization. This prompts the search for new and more effective methods of combating it. Thus, searching for and conducting new research on drugs that improve gastrointestinal motility seems reasonable.
